# Fixed point iteration for a countable family of multi-valued strictly pseudo-contractive-type mappings

**DOI:** 10.1186/s40064-015-1280-4

**Published:** 2015-09-17

**Authors:** C. E. Chidume, M. E. Okpala

**Affiliations:** Mathematics Institute, African University of Sciences and Technology, Abuja, Nigeria; Department of Mathematics, Federal University Ndufu-Alike Ikwo, Abakaliki, Ebonyi State Nigeria

**Keywords:** Generalized *k*-strictly pseudo-contractive multi-valued mappings, Multi-valued maps, 47H04, 47H09, 47H10

## Abstract

This paper introduces a new averaged algorithm for finding a common fixed point of a countably infinite family of generalized *k*-strictly pseudocontractive multi-valued mappings. The new iterative sequence introduced is proved to be an approximating fixed point sequence for common fixed points of a countably infinite family of this class of mappings. Furthermore, under some mild assumptions, strong convergence theorems are also proved for this class of mappings. The method of proof used here is new and enables to overcome many strong restrictions appearing in contemporary literature. The stated theorems improve and generalize many recent works in iterative scheme for multi-valued mappings.

## Background

Let (*X*, *d*) be a metric space, *K* a nonempty subset of *X*, and $T:K\rightarrow 2^K$ be a multi-valued mapping. A vector $x\in K$ is a fixed point of *T* if $x\in Tx$. For a single valued mapping *T*, a fixed point is any $x\in K$ such that $Tx=x$. We denote the collection of all fixed points of *T* by *F*(*T*). Many well known researchers like Brouwer (1912), Daffer and Kaneko (1995), Deimling (1992), and Kirk Downing and Kirk (1977), Geanakoplos (2003), Kakutani (1941), Markin (1973), Nadler (1969), Nash (1950, 1951) and Reich and Zaslavski (2002a, b, 2006), have studied fixed points for multi-valued mappings.

Fixed point theory for multi-valued mappings continues to attract a lot of attention because of its numerous real world applications in game theory and market economy, differential inclusions, and constrained optimization. They are also desirable in devising critical points in optimal control problems, energy management problems, signal processing, image reconstruction and a host of other problems.

Game theory and market economy is, perhaps, the most socially recognized application of multi-valued mappings.

Consider, for example, a game $G(x_n, K_n)$ involving *N* players, namely $n=1,2,\ldots ,N.$ Here, $K_n$, a nonempty compact and convex subset of $\mathbb {R}^{m_n}$, is the collection of possible strategies of the *n*th player. The continuous function $x_n:\Pi _{i=1}^NK_n\rightarrow \mathbb {R},$ is the gain(payoff) function. Any vector $y_n$ in $K_n$ is the action which is available to the individual *n* to take. The collective action of all the *N* players is then $y:=(y_1, y_2,\ldots ,y_N)\in K:=\Pi _{i=1}^NK_n.$ Given any *n*, *y* and $y_n\in K_n$, we use these standard notations:

$$\begin{aligned} K_{-n}:&=K_1\times K_2\times \cdots \times K_{n-1}\times K_{n+1}\times \cdots \times K_n\\ y_{-n}:&=(y_1, \ldots ,y_{n-1}, y_{n+1}, \ldots ,y_N)\\ (y_n,y_{-n})&=(y_1,y_2,\ldots ,y_{n-1},y_n,y_{n+1},\ldots ,y_N). \end{aligned}$$

In this regard, the $n'$th player maximizes his own gain, using a strategy $y_n^*$, subject to the fact that the other players have chosen their strategies $y_{-n}$ if and only if

$$\begin{aligned} x_n(y_n^*,y_{-n})=\max \limits _{y_n\in K_n}x_n(y_n,y_{-n}). \end{aligned}$$

Define a multi-valued mapping $T_n:K_{-n}\rightarrow 2^{K_{n}}$ by

$$\begin{aligned} T_n(y_{-n})=Arg\max \limits _{y_n\in K_n}x_n(y_n, y_{-n}) \end{aligned}$$

Then, the collective action $y^*=(y_1^*, y_2^*, \ldots ,y_N^*)$ is called a *Nash equilibrium* point if each $y_n^*$ is the most effective response that the $n'$th player can make to the actions $y_{-n}^*$ of the other $N-1$ players. This is stated differently as

$$\begin{aligned} x_n(y_n^*)=\max _{y_n\in K_n}x_n(y_n,y_{-n}^*), \end{aligned}$$

or, in other words,

$$\begin{aligned} y_n^*\in T_n(y_{-n}^*). \end{aligned}$$

Therefore, $y^*=(y_1^*, y_2^*,\ldots ,y_N^*)$ is a fixed point of the multi-valued mapping $T:K\rightarrow 2^K$ given by

$$\begin{aligned} T(y)=[T_1(y_{-1}),T_2(y_{-2}), \ldots ,T_N(y_{-N})]. \end{aligned}$$

Though many theory for multi-valued mappings in the literature have dealth with the existence of fixed points for such mappings, only very few have dealth with iterative algorithms for computing them. The problem of how to find such fixed points is part of what is addressed in this paper.

Given a real Hilbert space *H*, we denote by *CB*(*H*) the family of nonempty, closed and bounded subsets of *H*. It is well known that the Hausdorff distance defined by

$$\begin{aligned} D(A,B) := \max \limits \Big \{ \sup \limits _{a\in A} d(a,B), \sup \limits _{b\in B} d(b,A) \Big \}, \end{aligned}$$

is a metric on this family *CB*(*H*).

The first work on fixed points for multi-valued (nonexpansive) mappings by the application of Hausdorff metric was done by Markin (1973), and followed by an extensive work by Nadler (1969). Since then, there are many results that have appeared in the literature and which have found novel applications in both pure and applied sciences. Notable among these results is the work of Browder (1967).

In studying the operator equation $Au=0$ (when the mapping *A* is monotone), Browder (1967), introduced a new operator *T* defined by $T:=I-A,$ where *I* is the identity mapping on the Hilbert *H*. He called the operator a *pseudocontractive mapping* and the solutions of $Au=0$, are exactly the fixed points of the pseudocontractive mapping *T*. An important proper subclass of the pseudocontractive mappings is the well know nonexpansive mappings.

### **Definition 1.1**

A single-valued mapping $T:K\subseteq H\rightarrow H$ is called

- pseudo-contractive, in the terminology of Browder and Petryshyn (1967), if there exists $k\in [0,1)$ such that
  1 $$\begin{aligned} \Vert Tx-Ty\Vert ^2\le \Vert x-y\Vert ^2+k\Vert (x-Tx)-(y-Ty)\Vert ^2,\quad \forall x,y\in K. \end{aligned}$$
- monotone if
  $$\begin{aligned} \langle Tx-Ty, x-y\rangle \ge 0,\quad \forall x,y\in D(T). \end{aligned}$$

The class of pseudocontractive mappings is particularly important due to this close connection it has with the well known class of monotone mappings. Fixed points of the pseudocontractive mapping *T* are zeroes of the monotone mapping $A=I-T$. A well known example of a monotone operator in optimization theory is the multi-valued mapping $\partial f:D(f)\subseteq H\rightarrow 2^H$ called the subdifferential of the functional *f* and defined by

$$\begin{aligned} \partial f(x):=\{x^*\in X^*: \langle x-y, x^*\rangle \le f(x)-f(y), \,\, \forall y\in X\}. \end{aligned}$$

The theory of multi-valued nonexpansive mappings(and, in particular, pseudocontractive mappings) is much harder than the corresponding theory of single valued nonexpansive mappings [see e.g. Khan and Yildirim (2012)]. The extension of the notion of single valued pseudocontractive mappings to multi-valued pseudocontractive mappings has some of these challenges:

- *Definition of the mapping* There is a problem of getting a right definition for the multi-valued analogue which would be a generalization of the single-valued case. There are several definitions available which will be a generalisation of the single valued case and one has to get the most natural among them to be able to establish some convergence theorems.
- *Identities* In multi-valued settings, the metric induced by the norm on *X* is not applicable and there is the need to develop new identities and other notions of distances which will be applicable. One notion of metric for sets that is readily applicable here is the Hausdorf metric.
- *Inference* Many theorems and lemmas that are developed for single valued mappings cannot be carried over to multi-valued cases and it is always difficult to make conclusions.

Chidume et al. (2013), introduced a multi-valued analogue of Definition 1.2 as follows;

### **Definition 1.2**

Let *H* be a real Hilbert space and let *D* be a nonempty, open and convex subset of *H*. Let $T:\overline{D}\rightarrow CB(\overline{D} )$ be a mapping. Then, *T* is called *a multi-valuedk*-*strictly pseudocontractive mapping* if there exists $k\in (0,1)$ such that for all $x,y\in D(T)$, we have

2 $$\begin{aligned} D^2(Tx,Ty)\le \Vert x-y\Vert ^2+k\Vert (x-u)-(y-v)\Vert ^2 , \end{aligned}$$

for all $$u\in Tx, \,\,\,v\in Ty$$.

They proved a convergence theorem for this class of mapping as stated below:

### **Theorem 1.3**

(Chidume et al. (2013)) *LetKbe a nonempty, closed and convex subset of a real Hilbert space H*. *Suppose that*$T:K\rightarrow CB(K)$* is a multi-valued k-strictly pseudo-contractive mapping such that*$F(T)\ne \emptyset$. *Assume that*$Tp=\{p\}$*for all*$p\in F(T)$. *Suppose that T is hemicompact and continuous. Let*$\{x_n\}$*be a sequence defined iteratively from*$x_0\in K$*by*

3 $$\begin{aligned} x_{n+1}=(1-\lambda )x_n + \lambda y_n, \end{aligned}$$

*where*$y_n\in Tx_n$*and*$\lambda \in (0,1-k)$. *Then,*$\lim _{n\rightarrow \infty } d(x_n, Tx_n)=0$.

The result of Chidume et al. (2013) is more interesting than other similar result in the literature because it deals with strictly pseudocontractive mappings(which is more general than nonexpansive mappings) and also the problem of finding $z_n\in {Tx_n}$ such that $\Vert z_n-x^*\Vert =d(x^*,Tx_n)$ as it is, for example, in Sastry and Babu (2005), does not arise. However, the inequality (2) is equivalent to

4 $$\begin{aligned} D^2(Tx,Ty)&\le \Vert x-y\Vert ^2+k\inf \limits _{(u,v)\in (Tx,Ty)}\Vert (x-u)-(y-v)\Vert ^2. \end{aligned}$$

which is very restrictive

Very recently, Chidume and Okpala (2014) introduced a different class of multi-valued strictly pseudocontractive mapping as given below:

### **Definition 1.4**

Chidume and Okpala (2014) Let *H* be a real Hilbert space and let *K* be a nonempty subset of *H*. Let $T:K\rightarrow CB(K )$ be a multi-valued mapping. Then *T* is called *generalizedk*-*strictly pseudocontractive multi-valued mapping* if there exists $k\in (0,1)$ such that for all $x,y\in D(T)$, there holds

5 $$\begin{aligned} D^2(Tx,Ty)\le \Vert x-y\Vert ^2+kD^2(Ax,Ay), \quad \text {where} \quad A:=I-T, \end{aligned}$$

and *I* is the identity operator on *K*.

The class of mapping introduced here is natural and has been proved to be a proper superset of the class introduced in Chidume et al. (2013).

They developed some new identities regarding Hausdorf metric and used a Krasnoselskii type algorithm and obtained the following theorem.

### **Theorem 1.5**

(Chidume and Okpala (2014)) *Let K be a nonempty, closed, convex subset of a real Hilbert space H. Let*$T:K\rightarrow CB(K)$* be a generalized k-strictly pseudocontractive multi-valued mapping such that*$F(T)\ne \emptyset$. *Assume*$Tp=\{p\} \,\,\, \forall p\in F(T).$*Define a sequence*$\{x_n\}$*by*$x_0\in K$,

6 $$\begin{aligned} x_{n+1}=(1-\lambda )x_n+\lambda y_n \end{aligned}$$

*for*$y_n \in U^n$*and*$\lambda \in (0,1-k)$. *Then,*$d(x_n, Tx_n)\rightarrow 0$*as*$n\rightarrow \infty ,$*where*

$$\begin{aligned} U^n:=\Big \{y_n\in Tx_n:D^2(\{x_n\}, Tx_n)\le \Vert x_n-y_n\Vert ^2+\frac{1}{n^2}\Big \}. \end{aligned}$$

We seek to prove strong convergence theorems, using a new averaged algorithm, for common fixed point of a countably infinite family of this general class of mappings in a real Hilbert space. Our theorem generalizes the results of Chidume et al. (2013), Chidume and Ezeora (2014), Panyanak (2007), Song and Wang (2008), among others and extends to a countable family the results of Chidume and Okpala (2014).

## Preliminaries

We shall need the following definitions and notations in the sequel:

We casually denote $(D(A,B))^2$ by $D^2(A,B)$ for all $A, B\in CB(X)$ for simplicity of notation.

### **Definition 2.1**

A multi-valued mapping $T:K\subseteq H\rightarrow CB( H)$ is called

- Lipschitzian if there exists $L>0$ such that for each $x,y\in K$,
  7 $$\begin{aligned} D(Tx,Ty)\le L \Vert x-y\Vert , \end{aligned}$$
- nonexpansive if there exist $L\le 1$ such that *T* is Lipschitchitzian.

### **Proposition 2.2**

(Chidume and Okpala (2014)) *Let K be a nonempty subset of a real Hilbert space H and*$T:K\rightarrow CB(K)$*be a generalized k-strictly pseudocontractive multi-valued mapping. Then T is Lipschitzian.*

### *Remark 2.3*

Since every Lipschitz map is continuous, we would not make any continuity assumption on our mapping *T* throughout this paper.

### **Definition 2.4**

A map $T:K\rightarrow CB(K)$ is said to be *hemicompact* if, for any sequence $\{x_n\}$ such that $\lim \limits _{n\rightarrow \infty }d(x_n, Tx_n)=0,$ there exists a subsequence, say, $\{x_{n_k}\}$ of $\{x_n\}$ such that $x_{n_k}\rightarrow p\in K$.

### *Remark 2.5*

Trivial example of hemicompact mappings are mapping with compact domains.

### **Definition 2.6**

Let *H* be a real Hilbert space and let *T* be a multi-valued mapping. The multi-valued mapping $I-T$ is said to be *strongly demiclosed* at 0 (see, e.g., Garcí a-Falset et al. (2011)) if for any sequence $\{x_n\}\subseteq D(T)$ such that $x_n\rightarrow p$ and $d(x_n, Tx_n)$ converges strongly to 0, then $d(p, Tp)=0$.

### **Proposition 2.7**

(Chidume and Okpala (2014)) *Let K be a nonempty and closed subset of a real Hilbert space H and let*$T:K\rightarrow CB(K)$*be a generalized k-strictly pseudocontractive multi-valued mapping. Then,*$(I-T)$*is strongly demiclosed at zero.*

The following recurrent inequality will be used to make estimates in the sequel.

### **Lemma 2.8**

(Tan and Xu (1993)) *Let*$\{a_n\}$*be a sequence of nonnegative real numbers satisfying the following relation:*

$$\begin{aligned} a_{n+1}\le a_n+\sigma _n, \,\,\,n\ge 0, \end{aligned}$$

*such that*$\sum \nolimits _{n=1}^\infty \sigma _n<\infty .$*Then,*$\lim a_n$*exists. If, in addition,*$\{a_n\}$*has a subsequence that converges to 0, then*$a_n$*converges to 0 as*$n\rightarrow \infty .$

### **Lemma 2.9**

 (Chidume and Ezeora (2014)) *Let H be a real Hilbert space and let*$\{x_i, i=1,2,\ldots ,m\}\subseteq H$. *For*$\alpha _i\in (0,1), \,\,i=1,2,\ldots ,m$*such that*$\sum \nolimits _{i=1}^m\alpha _i=1,$*the following identity holds:*

$$\begin{aligned} \left\| \sum \limits _{i=1}^m\alpha _ix_i\right\| ^2=\sum \limits _{i=1}^m\alpha _i\Vert x_i\Vert ^2-\sum \limits _{1\le i<j\le m}\alpha _i\alpha _j\Vert x_i-x_j\Vert ^2, \end{aligned}$$

The following characterizations of the Hausdorf metric can be found in Chidume and Okpala (2014).

### **Lemma 2.10**

(Chidume and Okpala (2014)) *Let E be a normed linear space,*$B_1, B_2\in CB(E)$*and*$x, y\in E$*arbitrary. The following hold;*

- $$D(B_1, B_2)=D(x+B_1, x+B_2).$$*Translation Invariance.*
- $$ D(B_1, B_2)=D(-B_1,-B_2).$$
- $$ D(x+B_1, y+B_2)\le \Vert x-y\Vert +D(B_1,B_2).$$*Triangle inequality.*
- $$D(\{x\},B_1)=\sup \limits _{b_1\in B_1}\Vert x-b_1\Vert .$$
- $$D(\{x\}, B_1)=D(0,x-B_1).$$

## Fixed point iterations

The example given below shows that this general class of *k*-strictly pseudocontractive mappings actually exists and properly contains the class studied by Chidume et al. (2013), Osilike and Isiogugu (2011), Panyanak (2007), and a host of other authors. For the example, we shall need the following lemma, which is easy to verify.

### **Lemma 3.1**

*Let a, b, c be real numbers such that*$0\le a\le bc$, $c>0$. *Then*

8 $$\begin{aligned} (a-b)^2\le b^2+\left( \frac{c-2}{c}\right) a^2. \end{aligned}$$

### *Remark 3.2*

By setting $c=4$ in the lemma above, we will recover Lemma (3.5) of Chidume and Okpala (2014).

### *Example 3.3*

Define a multi-valued mapping $T_i:l_2(\mathbb {R})\rightarrow CB(l_2(\mathbb {R}))$ by

9 $$\begin{aligned} T_ix:= {\left\{ \begin{array}{ll} \{y\in l_2: \Vert x+y\Vert \le \alpha _i\Vert x\Vert \}, \quad x\ne 0\\ \{0\}, \quad \quad x=0, \end{array}\right. } \end{aligned}$$

where $\alpha _i=\frac{7i}{3i-1}$, $i=1,2,\ldots ,.$ We obtain that

$$\begin{aligned} x-T_ix:= {\left\{ \begin{array}{ll} \{y\in l_2: \Vert y-2x\Vert \le \alpha _i\Vert x\Vert \},\quad x\ne 0 \\ \{0\},\quad \quad x=0 \end{array}\right. } \end{aligned}$$

Then, for arbitrary $x,y\in l_2(\mathbb {R})$, we compute as follows:

$$\begin{aligned} D(T_ix,T_iy)=\Vert x-y\Vert +\alpha _i\Big |\Vert x\Vert -\Vert y\Vert \Big |, \end{aligned}$$

and

$$\begin{aligned} D(x-T_ix,y-T_iy)=2\Vert x-y\Vert +\alpha _i\Big | \Vert x\Vert -\Vert y\Vert \Big |. \end{aligned}$$

Now, set

$$\begin{aligned} a:=D(x-T_ix, y-T_iy); \,\,\,\,b:=\Vert x-y\Vert . \end{aligned}$$

Then, $$a-b=D(T_ix,T_iy)$$ and

$$\begin{aligned} a&=2\Vert x-y\Vert +\alpha _i\Big | \Vert x\Vert -\Vert y\Vert \Big |\\&\le (2+\alpha _i)\Vert x-y\Vert . \end{aligned}$$

Now, for each *i*, set $2+\alpha _i=c_i=c$ in Lemma (3.1) above. We obtain the identity $\frac{c_i-2}{c_i}=\frac{\alpha _i}{2+\alpha _i},$ and by the same lemma, we have

$$\begin{aligned} D^2(T_ix,T_iy)\le \Vert x-y\Vert ^2+\frac{\alpha _i}{2+\alpha _i}D(x-T_ix,y-T_iy). \end{aligned}$$

Thus, each $T_i, i=1,2,\ldots$, is a generalized $\kappa _i$-strictly pseudo-contractive multi-valued mapping with $\kappa _i=\frac{\alpha _i}{2+\alpha _i}\in (0,1)$ and each $\kappa _i\le \kappa :=\frac{7}{13}$. Moreover, we have $p\in T_ip$ if and only if $p=0$. Thus, for $p\in \cap _{i=1}^\infty F(T_ip)$, $T_ip=\{p\}$.

The following Lemma would be used in the sequel.

### **Lemma 3.4**

*Let H be a real Hilbert space and let*$\{x_i\}_{i\in \mathbb {N}}$* be a bounded sequence in H. For*$\delta _i\in (0,1),$*such that*$\sum \nolimits _{i=1}^\infty \delta _i=1,$*the following identity holds:*

10 $$\begin{aligned} \left\| \sum \limits _{i=1}^\infty \delta _ix_i\right\| ^2=\sum \limits _{i=1}^\infty \delta _i\Vert x_i\Vert ^2-\sum \limits _{1\le i<j< \infty }\delta _i\delta _j\Vert x_i-x_j\Vert ^2. \end{aligned}$$

### *Proof*

Define $\delta _i(n):=(1-\sum \nolimits _{n+1}^\infty \delta _i)^{-1}\delta _i$ for each *n*. It is easily seen that $\sum \nolimits _{i=1}^n\delta _i(n)=1$ and that $\delta _i(n)\rightarrow \delta _i$ as $n\rightarrow \infty$. Moreover, by Lemma 2.9, we obtain that

$$\begin{aligned} \left\| \sum \limits _{i=1}^n\delta _i((n)x_i\right\| ^2=\sum \limits _{i=1}^n\delta _i(n)\Vert x_i\Vert ^2-\sum \limits _{1\le i<j< \le n}\delta _i(n)\delta _j(n)\Vert x_i-x_j\Vert ^2. \end{aligned}$$

Since the inequality is true for all natural numbers *n*, we pass to the limit on both sides and obtain the identity (10) as proposed. $\square$

Next, given a countably infinite family $\{T_i\}_{i\ge 1}$ of generalized $\kappa _i$-strictly pseudo-contractive multi-valued mappings and an arbitrary sequence $\{x_n\}$ subset of *K*,  denote by $\Gamma _n^i$ the set of inexact distal points of $x_n$ with respect to the set $T_ix_n$, i.e

$$\begin{aligned} \Gamma _n^i:=\Big \{\zeta _n^i\in T_ix_n:D^2(\{x_n\}, T_ix_n)\le \Vert x_n-\zeta _n^i\Vert ^2+\frac{1}{n^2}\Big \}. \end{aligned}$$

Obviously, $\Gamma _n^i$ is closed, convex and nonempty for each $n\ge 1$ due to Lemma (2.10)(d).

In particular, if $T_ix$ is assumed to be proximinal and bounded for each $x\in K$, then $T_ix_n$ has a vector, say $\eta _n^i$, of maximum norm, i.e.

$$\begin{aligned} \Vert x_n-\eta _n^i\Vert =\sup \limits _{\zeta _n^i\in T_ix_n} \Vert x_n-\zeta _n^i\Vert =: D(\{x_n\}, T_ix_n). \end{aligned}$$

In that case, it is certain that $\eta _n^i\in \Gamma _n^i$.

Based upon these analyses, we now prove our main theorem. We will assume henceforth that *K* is a nonempty, closed and convex subset of a real Hilbert space *H*.

### **Theorem 3.5**

*Let*$T_i:K\rightarrow CB(K)$*be a countably infinite family of generalized*$\kappa _i$*-strictly pseudocontractive multi-valued mappings such that for some*$\kappa \in (0,1),\,\,\, \kappa _i\in (0,\kappa ]$. *Assume that*$\cap _{i=1}^\infty F(T_i)\ne \emptyset$*and for*$p\in \cap _{i=1}^\infty F(T_i),\,\,\, T_ip=\{p\}$. *Define the sequence*$\{x_n\}$*recursively by*

11 $$\begin{aligned} {\left\{ \begin{array}{ll} x_0\in K, \,\,\, arbitrary,\\ \zeta _n^i\in \Gamma _n^i,\\ x_{n+1}=\delta _0x_n+\sum \limits _{i=1}^\infty \delta _i \zeta _n^i,\\ \delta _0\in (\kappa ,1), \,\,\, \sum _{i=0}^\infty \delta _i=1. \end{array}\right. } \end{aligned}$$

*Then, for each i*, $\lim \nolimits _{n\rightarrow \infty }d(x_n, T_ix_n)= 0.$

### *Proof*

We will first of all establish that the recursion formula $x_{n+1}:=\delta _0x_n+\sum \nolimits _{i=1}^\infty \delta _i \zeta _n^i$ in the algorithm (11) is well defined. Take $p\in \cap _{i=1}^\infty F(T_i)$ arbitrary. We have

$$\begin{aligned} \Vert x_n-\zeta _n^i\Vert&\le D(x_n, T_ix_n),\\&=D(x_n+p, p+T_ix_n). \end{aligned}$$

Therefore, we obtain by Lemma 2.10(c) that

$$\begin{aligned} \Vert x_n-\zeta _n^i\Vert&\le \Vert x_n-p\Vert +D(Tp, T_ix_n),\,\\&\le \Vert x_n-p\Vert +\frac{1+\sqrt{\kappa }}{1-\sqrt{\kappa }}\Vert x_n-p\Vert . \end{aligned}$$

As a matter of fact, we may apply the triangle inequality and take limits to obtain

$$\begin{aligned} \Vert \zeta _n^i\Vert \le K_n:=\Vert x_n\Vert +\frac{2}{1-\sqrt{\kappa }}\inf _{p\in F(T)} \Vert x_n-p\Vert . \end{aligned}$$

It follows then that

$$\begin{aligned} \Vert x_{n+1}\Vert&\le \delta _0\Vert x_n\Vert +\sum \limits _{i=1}^\infty \delta _i \Vert \zeta _n^i\Vert ,\\ \end{aligned}$$

and therefore

$$\begin{aligned} \Vert x_{n+1}\Vert \le \delta _0\Vert x_n\Vert +\sum \limits _{i=1}^\infty \delta _i K_n \le K_n. \end{aligned}$$

which shows that $x_{n+1}$ is well defined. We show the convergence of $\{x_n\}$ as follows:

$$\begin{aligned} \Vert x_{n+1}-p\Vert ^2&= \Vert \delta _0(x_n-p)+\sum \limits _{i=1}^\infty \delta _i (\zeta _n^i-p)\Vert ^2\\&=\delta _0\Vert x_n-p\Vert ^2+ \sum \limits _{i=1}^\infty \delta _i \Vert \zeta _n^i- p\Vert ^2-\sum \limits _{i=1}^\infty \delta _0\delta _i\Vert x_n-\zeta _n^i\Vert ^2-\sum \limits _{1\le i\le j\le \infty }\delta _i\delta _j\Vert \zeta _n^i-\zeta _n^j\Vert ^2\\&\le \delta _0\Vert x_n-p\Vert ^2+\sum \limits _{i=1}^\infty \delta _i D^2(T_ix_n, Tp)-\sum \limits _{i=1}^\infty \delta _0\delta _i\Vert x_n-\zeta _n^i\Vert ^2\\&\le \delta _0\Vert x_n-p\Vert ^2+\sum \limits _{i=1}^\infty \delta _i (\Vert x_n-p\Vert ^2+\kappa _iD^2(\{0\}, x_n-T_ix_n ))-\sum \limits _{i=1}^\infty \delta _0\delta _i\Vert x_n-\zeta _n^i\Vert ^2\\&=\sum \limits _{i=0}^\infty \delta _i \Vert x_n-p\Vert ^2+\sum \limits _{i=1}^\infty \delta _i \kappa _iD^2(\{x_n\},T_ix_n)-\sum \limits _{i=1}^\infty \delta _0\delta _i\Vert x_n-\zeta _n^i\Vert ^2\\ \end{aligned}$$

Since $\zeta _n^i\in \Gamma _n^i$, we obtain that

$$\begin{aligned} \Vert x_{n+1}-p\Vert&\le \sum \limits _{i=0}^\infty \delta _i \Vert x_n-p\Vert ^2+\sum \limits _{i=1}^\infty \delta _i \kappa (\Vert x_n-\zeta _n^i\Vert ^2+\frac{1}{n^2})-\sum \limits _{i=1}^\infty \delta _0\delta _i\Vert x_n-\zeta _n^i\Vert ^2\\&\le \Vert x_n-p\Vert ^2+\frac{\kappa }{n^2}-\sum \limits _{i=1}^\infty \delta _i(\delta _0-k) (\Vert x_n-\zeta _n^i\Vert ^2). \end{aligned}$$

This is summarised as:

12 $$\begin{aligned} \Vert x_{n+1}-p\Vert ^2&\le \Vert x_n-p\Vert ^2+\frac{\kappa }{n^2}-\sum \limits _{i=1}^\infty \delta _i(\delta _0-\kappa ) \Vert x_n-\zeta _n^i\Vert ^2, \end{aligned}$$

and therefore

13 $$\begin{aligned} \Vert x_{n+1}-p\Vert ^2&\le \Vert x_n-p\Vert ^2+\frac{\kappa }{n^2}. \end{aligned}$$

In accordance with Lemma (2.8), $\Vert x_n-p\Vert$ has a limit and thus $\{x_n\}$ is bounded. Also, from inequality (12), there holds:

$$\begin{aligned} \sum \limits _{i=1}^\infty \delta _i(\delta _0-\kappa ) \Vert x_n-\zeta _n^i\Vert ^2\le \Vert x_n-p\Vert ^2+\frac{\kappa }{n^2}-\Vert x_{n+1}-p\Vert ^2 \end{aligned}$$

and so for each $i\ge 1$,

$$\begin{aligned} \delta _i(\delta _0-\kappa ) \Vert x_n-\zeta _n^i\Vert ^2\le \Vert x_n-p\Vert ^2+\frac{\kappa }{n^2}-\Vert x_{n+1}-p\Vert ^2,\,\,\rightarrow 0 (\text {as } n\rightarrow \infty ), \end{aligned}$$

Taking limits on both sides as $n\rightarrow \infty$, we conclude that $\lim \nolimits _{n\rightarrow \infty }\Vert x_n-\zeta _n^i\Vert =0$. Using the fact that $d(x_n, T_ix_n)\le \Vert x_n-\zeta _n^i\Vert$, we get $\lim \nolimits _{n\rightarrow \infty }d(x_n, T_ix_n)=0$. $\square$

### **Corollary 3.6**

*Let*$$T_i:K\rightarrow CB(K)$$*be a countably infinite family of generalized*$$\kappa _i$$*-strictly pseudocontractive multi-valued mappings such that for some*$$\kappa \in (0,1), \,\,\kappa _i\in (0,\kappa ]$$. *Assume that*$$\cap _{i=1}^\infty F(T_i)\ne \emptyset $$*and suppose that for*$$p\in \cap _{i=1}^\infty F(T_i),$$$$T_ip=\{p\}$$. *Assume*$$T_{i_0}$$*is hemicompact for some*$$i_0.$$*Then, the sequence*$$\{x_n\}$$*defined by algorithm* (11) *converges strongly to a fixed point of T*.

### *Proof*

We already have that $\lim \limits _{n\rightarrow \infty }d(x_n, T_ix_n)=0$ due to Theorem (3.5). The mapping $T_{i_0}$ being hemicompact guarantees the existence of some subsequence, say $\{x_{n_k}\}$, of $\{x_n\}$ such that $x_{n_k}\rightarrow q$ as $k\rightarrow \infty$. Let $\zeta _{n_k}^i\in T_ix_{n_k}$ be such that $\Vert x_{n_k}-\zeta _{n_k}^i\Vert \le d(x_{n_k}, T_ix_{n_k})+\frac{1}{k}$. We estimate that

$$\begin{aligned} d(q,T_iq)&\le \Vert q-x_{n_k}\Vert +\Vert x_{n_k}-\zeta _{n_k}^i\Vert +d(\zeta _{n_k}^i,T_iq)\\&\le \Vert q-x_{n_k}\Vert +d(x_{n_k}, T_ix_{n_k})+\frac{1}{k}+D(T_ix_{n_k}, T_iq)\\&\le \Vert q-x_{n_k}\Vert +d(x_{n_k}, T_ix_{n_k})+\frac{1}{k}+\frac{1+\sqrt{\kappa }}{1-\sqrt{\kappa }}\Big \Vert x_{n_k}-q\Big \Vert . \end{aligned}$$

If we take limits on both sides when $k\rightarrow \infty$, we have $d(q,T_iq)=0$. Using the fact that each $T_iq$ is closed, we obtain that $q\in T_iq$ for each *i*, and therefore conclude that $q\in \cap _{i=1}^\infty T_iq$. Moreover, $x_{n_k}\rightarrow q$ as $n\rightarrow \infty$ gives $\Vert x_{n_k}- q\Vert \rightarrow 0$ as $n\rightarrow \infty .$ Thus, by Lemma (2.8) and inequality (13), we get $\lim \limits _{n\rightarrow \infty }\Vert x_n-q\Vert =0.$ Thus $\{x_n\}$ converges strongly to a fixed point *q* of *T* as claimed. $\square$

### **Corollary 3.7**

*Let*$T_i:K\rightarrow CB(K)$*be a countably infinite family of generalized*$\kappa _i$*-strictly pseudocontractive multi-valued mapping, with*$\cap _{i=1}^\infty F(T_i)\ne \emptyset$*and assume that for*$p\in \cap _{i=1}^\infty F(T_i)$, $T_ip=\{p\}$. *Then, the sequence*$\{x_n\}$*defined by Eq.* (11) *converges strongly to a fixed point of T*.

### *Proof*

Since *K* is compact, the mappings $T_i:K\rightarrow CB(K)$ is hemicompact. Thus, by Corollary (3.6), we have that $\{x_n\}$ converges strongly to some $p\in F(T)$. $\square$

### *Remark 3.8*

In comparison with Theorem 7.1.5 of Chidume and Ezeora (2014), Corollary 3.6 has these merits.

(i) We proved the theorem for a countably infinite family of a much larger class of mapping which is the generalized *k*-strictly pseudo-contractive multi-valued mappings.
(ii) We only needed just one of the maps to be hemicompact and not all of them.
(iii) We replaced the ‘strong condition’ $\delta _i\in (k,1)$ by a weaker condition $\delta _0\in (k,1)$.
(iv) The condition $\zeta _n^i\in \Gamma _n^i$ is more readily applicable than requiring that *Tx* is proximinal and weakly closed for each *x*, and then, computing $\zeta _n=P_{Tx_n}x_n$ at each iterative step.

## Conclusion

Our theorem and corollaries improve the convergence theorems for multi-valued nonexpansive mappings in Abbas et al. (2011), Chidume et al. (2013), Chidume and Ezeora (2014), Chidume and Okpala (2014), Khan and Yildirim (2012), Ofoedu and Zegeye (2010), Panyanak (2007), Sastry and Babu (2005), Song and Wang (2008), in the following sense:

(i) The class of mappings considered in this paper contains the class of multi-valued *k*-strictly pseudocontractive mappings as a special case, which itself properly contain the class of multi-valued nonexpansive maps.
(ii) The algorithm here is of Krasnoselkii type, which is known to have a geometric order of convergence.
(iii) The condition that *Tx* be weakly closed for each $x\in K$ as can be found, for example, in Chidume et al. (2013) and Chidume and Ezeora (2014) is dispensed with here.
